# Preoperative carbohydrate antigen 19.9 level predicts lymph node metastasis in resectable adenocarcinoma of the head of the pancreas: a further plea for biological resectability criteria

**DOI:** 10.1097/JS9.0000000000000773

**Published:** 2023-09-22

**Authors:** Alessandro Coppola, Vincenzo La Vaccara, Tommaso Farolfi, Horacio J. Asbun, Ugo Boggi, Kevin Conlon, Bjørn Edwin, Cristina Ferrone, Eduard Jonas, Norihiro Kokudo, Elena Martin Perez, Sohei Satoi, Ernesto Sparrelid, John Stauffer, Alessandro Zerbi, Nobuyuki Takemura, Quirino Lai, Tariq Almerey, Marc Bernon, Roberto Cammarata, Yasmine Djoumi, Tom Gallagher, Poya Ghorbani, Michael Ginesini, Daisuke Hashimoto, Emanuele F Kauffmann, Dyre Kleive, Núria Lluís, Rocio Maqueda González, Niccolò Napoli, Gennaro Nappo, Martina Nebbia, Simone Ricchitelli, Mushegh A. Sahakyan, Tomohisa Yamamoto, Roberto Coppola, Damiano Caputo

**Affiliations:** a Dipartimento di Chirurgia, Sapienza Università di Roma,; b General Surgery, Fondazione Policlinico Universitario Campus Bio-Medico; c General Surgery and Organ Transplantation Unit, Sapienza University of Rome, Umberto I Polyclinic of Rome, Rome; d Division of General and Transplant Surgery, University of Pisa, Pisa; e Pancreatic Surgery Unit, Humanitas Clinical and Research Center—IRCCS, Rozzano, Milan; f Department of Biomedical Sciences, Humanitas University, Pieve Emanuele, Italy; g Division of Hepatobiliary and Pancreas Surgery, Miami Cancer Institute, Miami; h Division of Surgical Oncology, Minimally Invasive and Hepatobiliary Surgery, Mayo Clinic in Florida, Jacksonville, FL, USA; i Department of HPB Surgery, St. Vincent’s University Hospital, Dublin, Ireland; j The Intervention Center; k Department of Research; Development, Division of Emergencies and Critical Care; l Department of HPB Surgery, Oslo University Hospital, Rikshospitalet; m Institute of Clinical Medicine, Medical Faculty, University of Oslo, Oslo, Norway; n Department of Surgery, Massachusetts General Hospital, Boston, MA, USA; o Department of Surgery, University of Cape Town Faculty of Health Sciences, Surgical Gastroenterology Unit, Groote Schuur Hospital, Cape Town, South Africa; p Hepato-Biliary Pancreatic Surgery Division, Department of Surgery, National Center for Global Health and Medicine,Toyama, Shinjyuku-ku, Tokyo; q Department of Surgery, Kansai Medical University, Hirakata City, Osaka, Japan; r General Surgery Department, La Princesa Hospital, Health Research Institute Princesa (IIS-IP), Autónoma de Madrid University (UAM), Madrid, Spain; s Department of Clinical Science, Intervention and Technology, Division of Surgery, Karolinska Institutet, Karolinska University Hospital, Stockholm, Sweden; t Department of Surgery N1, Yerevan State Medical University, Yerevan, Armenia

**Keywords:** CA 19.9, lymph node involvement, Pancreatic adenocarcinoma, pancreatic cancer markers, pancreatoduodenectomy, resectable pancreatic adenocarcinoma

## Abstract

**Introduction:**

Lymph-nodal involvement (N+) represents an adverse prognostic factor after pancreatoduodenectomy (PD) for pancreatic adenocarcinoma (PDAC). Preoperative diagnostic and staging modalities lack sensitivity for identifying N+. This study aimed to investigate preoperative carbohydrate antigen 19.9 (CA 19.9) in predicting the N+ stage in resectable-PDAC (R-PDAC).

**Methods:**

Patients included in a multi-institutional retrospective database of PDs performed for R-PDAC from January 2000 to June 2021 were analysed. A preoperative laboratory value of CA 19.9 greater than 37 U/l was used in univariate and multivariate logistic regression analysis to determine a possible association with N+. Additionally, different cut-offs of CA 19.9 related to the preoperative clinical T (cT) stage was assessed to evaluate the risk of N+.

**Results:**

A total of 2034 PDs from thirteen centres were included in the study. CA 19.9 greater than 37 U/l was significantly associated with higher N+ at univariate and multivariate analysis (*P*<0.001). CA 19.9 levels greater than 37 U/l were associated with N+ in 75.9%, 81.3%, and 85.7% of patients, respectively, in cT1, cT2, and cT3 tumours and with higher cut-off values for all cT stages.

**Conclusion:**

Lymph-nodal involvement is strongly related to preoperative CA 19.9 levels. Specially in patients staged as cT3 the CA 19.9 could represent a valid and easy tool to suspect nodal involvement. Due to these findings, R-PDAC patients with elevated CA 19.9 values should be considered in a more biologically advanced stage.

## Introduction

Highlights

- This is a retrospective multicenter study including a total of 2034 patients underwent upfront pancreatoduodenectomy for pancreatic ductal adenocarcinoma.
- Primary endpoint was to define the relationships between preoperative carbohydrate antigen 19.9 (CA 19.9) levels and the presence of lymph node metastasis at final histology; secondary endpoint of this study was to define a cut-off value of CA 19.9 that accurately predicts the presence of lymph node metastasis.
- The present study showed that the standard laboratory cut-off value of CA 19.9 (i.e. 37 U/ml) played a role in the prediction of nodal involvement, elevations of CA 19.9 levels above the standard laboratory cut-off are significantly associated with a high rate of nodal involvement (>80%).
- At multivariable analysis, CA 19.9 was confirmed to be an independent risk factor for nodal involvement; more in detail every logarithmic increase of CA 19.9 resulted in doubling the odds of nodal positivity.
- In light of our findings, we can postulate that the presence of resectable-pancreatic adenocarcinoma (R-PDAC) with CA 19.9 greater than 37 U/ml preoperative staging may suggest a shift from a “radiologically-related” upfront resectable status to a “biologically-related” borderline resectable status.
- A sub-analysis was performed in different classes of bilirubin, showing no impact of the different cholestasis severity strata in terms of lymph node positivity discrimination.

Pancreatic cancer (PDAC) remains the fourth leading cause of death among all malignancies, with increasing incidence in both women and men^1^. Surgical resection is considered the gold standard curative option for PDAC. Unfortunately, up to 80% of patients are not eligible for surgery at the time of diagnosis due to locally advanced disease or systemic spread^2,3^.

Preoperative assessment of biological resectability is gaining momentum instead of still widely used anatomical resectability criteria^4^. A consensus meeting of the International Association of Pancreatology (IAP) introduced several biological factors, including carbohydrate antigen 19.9 (CA 19.9) level, in the definition of borderline resectable-PDAC (R-PDAC) independent from anatomical characteristics^5^.

Moreover, loco-regional lymphadenopathy detected at staging is considered a worrisome feature^6^. Lymph node involvement significantly impacts the survival of patients with upfront resected PDAC^7^. It is suggested that anatomically R-PDAC with suspicious biological features should be considered and treated as borderline resectable PDAC (BR-PDAC).

Unfortunately, preoperative confirmation of metastatic lymph nodes is suboptimal with standard imaging investigations. Several reports highlighted on using MRI, computed tomography, or radiomics to increase the preoperative diagnostic rates of lymph node metastases; however, these radiological tools are nowadays related only to dimensional characteristics^8–10^.

The role of CA 19.9 at different cut-offs in predicting lymph node involvement, or margin status after surgery, is primarily debated in the literature with conflicting results^11,12^.

We hypothesised that R-PDAC patients with radiologically negative nodes and higher preoperative CA 19.9 values who undergo radical surgery would be found to have higher rates of positive nodes at pathology.

This study aimed to compare the rates of pathologically positive nodes in two groups of R-PDAC patients with preoperative high or low CA 19.9 values in an international patient cohort.

## Materials and methods

### Study design

In this retrospective international multicentre study, participating centres entered data on upfront pancreatoduodenectomy (PD) for R-PDAC performed between 1 January 2000, and 30 June 2021 into the “International Validation of CA 19.9 Serum Level in the Prediction of Lymph-Nodes Status in Resectable Pancreatic Ductal Adenocarcinoma in Normo-Albumin Serum Levels Patient (ICALYRA)” project database.

The study protocol was approved by the Ethical Committee of the coordinator centre (PAR 119/21 (OSS)), and the Local Ethics Boards of all the involved centres. The Strengthening the Reporting of Observational Studies in Epidemiology (STROBE) guidelines were followed to create the study. The study has been reported in line with the Strengthening The Reporting Of Cohort Studies in Surgery (STROCSS) criteria, Supplemental Digital Content 1, http://links.lww.com/JS9/B80 ^13^.

### Population

Patients aged older than or equal to 18 years with R-PDAC undergoing upfront PD for PDAC, with no preoperative radiological suspicion of lymph node metastases.

### Endpoints

The primary endpoint of this study was to define the relationships between preoperative CA 19.9 levels and the presence of lymph node metastasis at final histology.

The secondary endpoint of this study was to define a cut-off value of CA 19.9 that accurately predicts the presence of lymph node metastasis.

### Data collection

Data were retrospectively collected from prospective institutional databases of each participating centre. The guarantor of the data quality was the data manager of the study group (A.C.). Data errors and missingness were identified across the database and solved, when possible, with specific queries.

### Definitions

The cut-off value of CA 19.9 to differentiate between high- and low-value patients corresponded to the internationally recognised normal value of 37 U/ml^14^.

The CA 19.9 and bilirubin values reported in the database were obtained during the same preoperative diagnostic phase. In other words, the CA 19.9 value corresponds to the bilirubin value recorded for each patient at that specific moment.

R-PDACs were defined as pancreatic head tumours with (a) no preoperative radiological evidence of portal vein and superior mesentery vein involvement, (b) clear fat planes around the coeliac trunk, hepatic artery, and superior mesentery artery, and (c) no distant metastases^4,6–12^.

BR-PDAC were defined as pancreatic PDAC with venous involvement of the superior mesenteric vein or portal vein with distortion or narrowing of the vein or occlusion of the vein with suitable vessel proximal and distal, allowing for safe resection and replacement. No tumour contact/invasion of superior mesenteric artery, coeliac trunk or common hepatic artery^4,5^.

Pathology reporting followed the Union for International Cancer Control (UICC) staging system, 8^th^ edition^15^.

To be included, patients should have undergone at least a standard lymphadenectomy for pancreatoduodenectomy as described by the International Study Group on Pancreatic Surgery (ISGPS)^16^.

Positive margins (R1) were defined as tumours located less than or equal to 1 mm from the resection margin.

Patients diagnosed with distant metastases during the preoperative workup or surgical exploration were not included in the database.

### Statistical analysis

Continuous variables were reported as medians and 1st–3rd quartiles (Q1–Q3). Categorical variables were described as numbers and percentages. Comparisons between groups were made using Fisher’s exact test or the ^χ^ test for categorical variables, as appropriate. The Mann–Whitney test was used for continuous variables. Missing data involved less than 10% of patients (SM-Table 1, Supplemental Digital Content 2, http://links.lww.com/JS9/B81). Missing data were handled with a single imputation method using a median of nearby points imputation. The median instead of the mean was adopted for skewed distribution of the managed variables.

Multivariable logistic regression analysis was run to identify the risk factors for nodal positivity after surgery. The investigated variables were selected using a ‘full model’ approach. A backward Wald method was adopted for constructing the final model. Odds ratios (OR) and 95% CI were reported for significant variables.

Only preoperatively available variables were included in this multivariable analysis model with the intent to avoid potential immortal bias phenomena in the construction of the model.

The predictive ability of CA 19.9 level for diagnosing nodal positivity was performed. The area under the curve (AUC) and 95% CI were reported. The diagnostic OR (DOR) value was also evaluated for different CA 19.9 cut-off values, corresponding to previous values reported in the literature^14^.

Variables with a *P* less than 0.05 were considered statistically significant. Statistical analyses and plots were run using the SPSS statistical package version 27.0 (SPSS Inc.).

## Results

A total of 2034 patients were included in the study. Patient characteristics are summarised in Table 1. The population was divided into two groups according to the preoperative CA 19.9 value: the low-value group (LVG; *n* = 596, 29.3%), and the high-value group (HVG; *n* = 1438, 70.7%) (Table 1).

**Table 1:** Characteristics of the investigated population and in the two groups of patients with high and low CA 19.9.

		Low-value CA 19.9 (*n*=596, 29.3%)	High-value CA 19.9 (*n*=1438, 70.7%)	
Variables	Entire cohort (*n*=2034)	Median (1st–3rd Q) or *n* (%)		*p*
Age, years	70 (63–76)	70 (63–76)	70 (62–76)	0.72
Male sex	1057 (51.9)	312 (52.3)	745 (51.8)	0.85
BMI ≥30	179 (8.8)	46 (7.7)	133 (9.2)	0.30
T2DM	566 (27.8)	150 (25.2)	416 (28.9)	0.09
ASA 3–4	1029 (50.5)	294 (49.3)	735 (51.1)	0.47
Preoperative CA 19.9, U/l	116.1 (30.0–501.0)	12.4 (4.3–24.0)	255.6 (98.4–860.5)	<0.001
Preoperative albumin, mg/dl	3.6 (3.3–4.1)	3.8 (3.3–4.1)	3.6 (3.2–4.0)	<0.001
Preoperative total bilirubin, mg/dl	1.4 (0.7–4.0)	1.0 (0.6–2.7)	1.6 (0.7–4.9)	<0.001
Radiological tumour size, mm	27 (20–35)	25 (20–35)	28 (22–35)	<0.001
Radiological T (UICC 8 ed)				
T1	540 (26.6)	208 (34.9)	332 (23.1)	<0.001
T2	1276 (62.7)	321 (53.9)	955 (66.4)	
T3	218 (10.7)	67 (11.2)	151 (10.5)	
Pathological tumour size, mm	30 (25–40)	30 (22–37)	32 (25–40)	<0.001
Pathological T (UICC 8 ed)				
T1	306 (15.1)	130 (21.8)	176 (12.2)	<0.001
T2	1300 (63.9)	362 (60.7)	938 (65.2)	
T3	396 (19.4)	94 (15.8)	302 (21.0)	
T4	32 (1.6)	10 (1.7)	22 (1.5)	
Pathological N (UICC 8 ed)				
N0	458 (22.6)	193 (32.4)	265 (18.4)	<0.001
N1	755 (37.2)	209 (35.1)	546 (38.0)	
N2	821 (40.3)	194 (32.6)	627 (43.6)	
Harvested nodes, *n*	26 (18–37)	27 (18–38)	26 (18–37)	0.16
Positive nodes, *n*	3 (1–6)	2 (0–5)	3 (1–6)	<0.001
Ratio of nodes positive/harvested, %	10 (0–20)	10 (0–20)	10 (0–30)	<0.001
Margin Status				
R1	833 (40.9)	199 (33.4)	634 (44.1)	<0.001
R2	7 (0.3)	3 (0.5)	4 (0.3)	
Tumour grading				
G1	181 (8.9)	78 (13.1)	103 (7.2)	<0.001
G2	1,066 (52.4)	318 (53.4)	748 (52.0)	
G3	512 (25.2)	142 (23.8)	370 (25.7)	
G4	255 (12.5)	54 (9.1)	201 (14.0)	
No grading evaluable	20 (1.0)	4 (0.7)	16 (1.1)	

ASA, American Society of Anesthesiologists; CA 19.9, carbohydrate antigen 19.9; *n*, number; Q, quartile; T2DM, type-2 diabetes mellitus; UICC, Union for International Cancer Control.

HVG patients had a significantly higher median level of total bilirubin (1.6 vs. 1.0 mg/dl; *P*<0.001), a larger median tumour diameter at radiology (28 vs. 25 mm; *P*<0.001), and a higher median value of CA 19.9 (255.6 vs. 12.4 U/ml; *P*<0.001). Preoperative median albumin levels were significantly higher in the LVG patients (3.8 vs. 3.6 mg/dl; *P*<0.001). No differences were observed between the two groups regarding age, sex, BMI, or diabetes.

The pathological reports confirmed the data showed by the preoperative radiological staging, with a larger median tumour size in the HVG patients (32 vs. 30 mm; *P*<0.001). The pathological T stage showed a significant difference, with the T1–T2 stage reported in 82.5 vs. 77.4% in LVG vs. HVG patients, respectively (*P*<0.001).

The number of harvested lymph nodes was similar in the two groups, with a median number of 27 (Q1–Q3 = 18–38) in the LVG and 26 (Q1–Q3 = 18–37) in the HVG (*P*=0.16). The N stage was statistically different between the two groups (*P*<0.001), with an N0 reported in 32.4% vs. 18.4% in LVG versus HVG patients, respectively. Two-hundred nine (35.5%) LVG patients and 546 (38.0%) HVG patients were classified as N1. Lastly, the N2 stage was present in 194 (32.6%) LVG patients and 627 (43.6%) HVG patients.

One-hundred ninety-nine (33.4%) LVG patients and 634 (44.1%) HVG patients had R1 (*P*<0.001). In addition, three (0.5%) LVG patients and four (0.3%) HVG patients were classified as R2.

High and low tumour grades were reported in 370 (25.7%) and 201 (14.0%) HGV patients versus 142 (23.8%) and 54 (9.1%) LVG patients (*P*<0.001).

### Risk factors for positive lymph nodes at pathology

At multivariable logistic regression analysis, log10 CA 19.9 (OR = 1.41, 95% CI = 1.26–1.59; *P*<0.001) and log10 total bilirubin (OR = 1.29, 95% CI = 1.03–1.61; *P*=0.03) correlated with positive pathological N status (Table 2). Patient age (OR = 0.98, 95% CI= 0.97–0.98; *P*<0.001) and albumin levels (OR = 0.74, 95% CI = 0.61–0.99; *P*=0.002) correlated with statistically significant decreased odds. The radiological tumour dimension did not show any statistical relevance. A separate analysis of risk factors for pathological N2 status showed a positive correlation with log10 CA 19.9 (OR = 1.36, 95% CI = 1.23–1.50; *P*<0.001) and radiological tumour size (OR = 1.01, 95% CI = 1.01–1.02; *P*<0.001). Patient age (OR = 0.99, 95% CI = 0.98–0.99; *P*=0.001) and serum albumin levels (OR = 0.74, 95% CI = 0.64–0.87; *P*<0.001) also confirmed a potential protective role in the setting of pathological N2 positivity.

**Table 2:** Multivariable logistic regression analyses for the risk factors of positive nodes at pathology.

Variables	Beta	SE	Wald	OR	95.0% CI	*p*
Any pN status (*)						
Log10 CA 19.9, per 0.1 U/l	0.35	0.06	33.85	1.41	1.26–1.59	<0.001
Patient age, years	−0.02	0.006	11.74	0.98	0.97–0.99	<0.001
Albumin, per 0.1 g/dl	−0.30	0.10	9.26	0.74	0.61–0.90	0.002
Log10 total bilirubin, per 0.1 mg/dl	0.25	0.11	4.84	1.29	1.03–1.61	0.03
Constant	2.90	0.58	25.37	18.24	—	<0.001
pN2 status (**)						
Log10 CA 19.9, per 0.1 U/l	0.31	0.05	36.37	1.36	1.23–1.50	<0.001
Albumin, per 0.1 g/dl	−0.30	0.08	14.63	0.74	0.64–0.87	<0.001
Tumour size, per 1 mm	0.01	0.004	11.03	1.01	1.01–1.02	<0.001
Patient age, years	−0.02	0.005	10.51	0.99	0.98–0.99	0.001
Constant	0.66	0.47	1.97	1.94	-	0.16

Backward Wald method.

Hosmer-Lemeshow test: 0.18 (*); 0.91 (**).

Variables initially introduced into the mathematical models: patient age, patient BMI, T2DM, ASA 3–4, log10 CA 19.9, log10 total bilirubin, albumin, tumour size.

ASA, American Society of Anesthesiologists; CA 19.9, carbohydrate antigen 19.9; OR, odds ratio; SE, standard error; T2DM, type-2 diabetes mellitus.

### Lymph nodes positivity at pathology, preoperative CA 19.9 levels, and radiological T status

Results of a sub-analysis of five patient cohorts stratified according to the CA 19.9 values and the number of harvested nodes, number of positive nodes, the ratio of positive nodes, and N1 and N2 stages, are shown in Table 3.

**Table 3:** Pathologic lymph nodes positivity results according to clustered preoperative CA 19.9 levels in the entire population and in the sub-classes of patients according to the radiological T status.

CA 19.9 U/ml	*n*	N+	N1	N2	Harvested	Number of N+	Ratio N+/harvested, %
Entire population							
≤37	596	403 (67.6)	209 (35.1)	194 (32.6)	27 (18–38)	2 (0–5)	10 (0–20)
38–249	709	568 (80.1)	295 (41.6)	273 (38.5)	25 (17–36)	3 (1–5)	10 (0–20)
250–499	220	181 (82.3)	89 (40.5)	92 (41.8)	26 (17–36)	3 (1–6)	10 (0–30)
500–999	193	161 (83.4)	66 (34.2)	95 (49.2)	26 (19–38)	3 (1–7)	10 (0–30)
≥1000	316	263 (83.2)	96 (30.4)	167 (52.8)	26 (19–38)	4 (1–8)	10 (10–30)
*p* value	—	<0.001	<0.001	0.34	<0.001	<0.001	
cT1							
≤37	208	130 (62.5)	71 (34.1)	59 (28.4)	24 (17–34)	1 (0–4)	0 (0–20)
38–249	203	154 (75.9)	85 (41.9)	69 (34.0)	22 (16–33)	2 (1–5)	10 (0–20)
250–499	49	43 (87.8)	23 (46.9)	20 (40.8)	23 (17–33)	2 (1–6)	10 (0–35)
500–999	28	21 (75.0)	10 (35.7)	11 (39.3)	23 (16–33)	2 (0–7)	10 (0–28)
≥1000	52	39 (75.0)	21 (40.4)	18 (34.6)	23 (16–39)	2 (0–5)	10 (0–20)
*p* value	—	0.002	0.03	0.94	0.02	0.009	
cT2							
≤37	321	241 (75.1)	129 (40.2)	112 (34.9)	28 (18–38)	2 (1–5)	10 (0–20)
38–249	443	360 (81.3)	186 (42.0)	174 (39.3)	26 (18–38)	3 (1–6)	10 (0–20)
250–499	149	120 (80.5)	56 (37.6)	64 (43.0)	26 (17–36)	3 (1–6)	10 (0–30)
500–999	148	127 (85.8)	51 (34.5)	76 (51.4)	27 (20–38)	4 (2–8)	20 (10–30)
≥1000	215	181 (84.2)	61 (28.4)	120 (55.8)	26 (19–37)	4 (1–8)	20 (10–30)
*p* value	—	0.03	<0.001	0.44	<0.001	<0.001	
cT3							
≤37	67	32 (47.8)	9 (13.4)	23 (34.3)	29 (19–49)	0 (0–5)	0 (0–10)
38–249	63	54 (85.7)	24 (38.1)	30 (47.6)	25 (16–41)	3 (2–6)	10 (10–20)
250–499	22	48 (81.8)	10 (45.5)	8 (36.4)	37 (26–55)	3 (2–5)	10 (0–20)
500–999	17	13 (76.5)	5 (29.4)	8 (47.1)	28 (22–47)	3 (1–5)	10 (0–25)
≥1000	49	43 (87.8)	14 (28.6)	29 (59.2)	29 (20–45)	5 (3–8)	20 (10–30)
*p* value	—	<0.0001	<0.001	0.19	0.001	<0.001	

CA 19.9, carbohydrate antigen 19.9; cT1, radiological T1 stage; cT2, radiological T2 stage; cT3, radiological T3 stage; n, number; N+, nodal positivity at pathology; N1, nodal 1 stage; N2, nodal 2 stage.

In all the sub-classes with CA 19.9 greater than 37 U/ml, the percentage of N+ patients increased from 80.1 to 83.4%. The percentage of N2 patients also increased progressively with the increase of CA 19.9 values, with 52.8% of cases observed when CA 19.9 values were greater than 1000 U/ml. Interestingly, the number of harvested nodes was not statistically different in the different sub-classes.

A similar analysis was also performed according to the different radiological T stages (Table 3).

This percentage increased with increasing CA 19.9 levels and tumour diameter, with 87.8% being N+ and 59.2% being N2 observed in cT3 patients with CA 19.9 values greater than 1000 U/ml.

### Prediction of lymph nodes positivity

CA 19.9 showed a sufficient ability to predict the risk of positive lymph nodes (AUC = 0.60, 95% CI = 0.57–0.63; *P*<0.001) (Table 4). Testing different cut-offs, a value of 37 U/ml showed a sensitivity of 74.4% and a specificity of 42.1%, with the best DOR among the different threshold values tested (DOR = 2.1).

**Table 4:** Predictive ability of CA 19.9 for pN and pN2 positivity.

AUC (95.0% CI)	SE	*p*	CA 19.9 U/ml cut-off (centile)	Sensitivity	Specificity	DOR
pN positivity (pN1 plus pN2)						
0.60 (0.57–0.63)	0.02	<0.001	37 (29th)	74.4	42.1	2.1
			250 (64th)	38.4	72.9	1.7
			500 (75th)	26.9	81.4	1.6
			1000 (85th)	16.8	88.4	1.5
			1827 (90th)	11.4	95.0	2.6
pN2						
0.59 (0.56–0.61)	0.01	<0.001	37 (29th)	76.7	33.1	1.6
			250 (64th)	43.1	69.1	1.7
			500 (75th)	31.9	79.6	1.8
			1000 (85th)	20.6	87.7	1.8
			2578 (92th)	11.4	95.0	2.4

AUC, area under the curve; CA 19.9, carbohydrate antigen 19.9; DOR, diagnostic odds ratio; pN1, pathological nodal 1 stage; pN2, pathological nodal 2 stage; SE, standard error.

Similar prediction abilities of CA 19.9 were observed for the N2 status (AUC = 0.59, 95% CI = 0.56–0.61; *P*<0.001) (Table 4). A cut-off of 37 U/ml had a sensitivity and specificity of 76.7% and 33.1%, respectively (DOR = 1.6).

A sub-analysis was performed, in which the prediction ability of CA 19.9 was estimated according to the initial radiological T stage (Table 5). In cT1 and cT2 patients, the diagnostic ability of CA 19.9 for an N+ status was poor-to-sufficient, with an AUC ranging from 0.57 to 0.60. In cT3 patients, the diagnostic ability increased (AUC = 0.71, 95% CI = 0.63–0.79; *P*<0.001). Using a cut-off of 37 U/ml, sensitivity and specificity were 80.0% and 60.3%, respectively (DOR = 6.1).

**Table 5:** Predictive ability of CA 19.9 for pN positivity. Population stratified for radiological T status.

AUC (95.0% CI)	SE	*p*	CA 19.9 U/ml cut-off (centile)	Sensitivity	Specificity	DOR
cT1						
0.60 (0.54–0.65)	0.03	0.001	37 (29th)	66.4	50.3	2.0
			250 (64th)	26.9	83.0	1.8
			500 (75th)	15.5	86.3	1.2
			1000 (85th)	10.1	91.5	1.2
cT2						
0.57 (0.53–0.61)	0.02	0.001	37 (29th)	76.7	32.0	1.5
			250 (64th)	41.7	66.0	1.4
			500 (75th)	29.9	77.7	1.5
			1000 (85th)	17.8	86.2	1.4
cT3						
0.71 (0.63–0.79)	0.04	<0.001	37 (29th)	80.0	60.3	6.1
			250 (64th)	46.3	75.9	2.7
			500 (75th)	35.6	82.8	2.7
			1000 (85th)	27.5	89.7	3.3

AUC, area under the curve; CA 19.9, carbohydrate antigen 19.9; cT1, radiological T1 stage; cT2, radiological T2 stage; cT3, radiological T3 stage; DOR, diagnostic odds ratio; SE, standard error.

Lastly, a similar sub-analysis was performed in which the diagnostic ability of CA 19.9 was estimated according to the post-operative pathological T stage (Table 6). The highest diagnostic ability was found in pT3 patients (AUC = 0.67, 95% CI = 0.60–0.75; *P*<0.001). Using a cut-off of 37 U/mL, sensitivity and specificity were 83.1% and 54.9%, respectively (DOR = 6.0).

**Table 6:** Population stratified for pathological T status. Risk for pN positivity.

AUC (95.0% CI)	SE	*p*	CA 19.9 U/ml cut-off (centile)	Sensitivity	Specificity	DOR
pT1						
0.57 (0.50–0.63)	0.03	0.050	37 (29th)	63.5	50.4	1.8
			250 (64th)	23.8	83.2	1.5
			500 (75th)	14.4	88.0	1.2
			1000 (85th)	7.7	93.6	1.2
pT2						
0.57 (0.53–0.61)	0.02	0.001	37 (29th)	73.9	34.1	1.5
			250 (64th)	38.0	69.0	1.4
			500 (75th)	26.8	79.5	1.4
			1000 (85th)	16.1	87.2	1.3
pT3						
0.67 (0.60–0.75)	0.04	<0.001	37 (29th)	83.1	54.9	6.0
			250 (64th)	49.5	70.4	2.3
			500 (75th)	35.1	78.9	2.0
			1000 (85th)	24.6	84.5	1.8
pT4						
0.42 (0.12–0.72)	0.16	0.61	37 (29th)	71.4	0.25	0.01
			250 (64th)	25.0	50.0	0.3
			500 (75th)	17.9	50.0	0.2
			1000 (85th)	14.3	50.0	0.2

AUC, area under the curve; CA 19.9, carbohydrate antigen 19.9; DOR, diagnostic odds ratio; pT1, radiological T1 stage; pT2, pathological T2 stage; pT3, pathological T3 stage; pT4, pathological T4 stage; SE, standard error.

### Analysis of potential confounding factors

Different cut-offs of CA 19.9 were additionally analysed at different bilirubin levels for the prediction of lymph node involvement. As shown in SM-Table 2, Supplemental Digital Content 3, http://links.lww.com/JS9/B82, the percentages of nodal involvement remain substantially similar in the different CA 19.9 strata no matter if the bilirubin levels were normal (≤ 1.50 mg/dl), borderline (1.51–3.00 mg/dl), or high (≥3.00 mg/dl).

An additional analysis was performed in CA 19.9 non-secretors, namely in the sub-cohort of patients with a CA 19.9 level less than or equal to 37 U/ml (*n* = 596). Dividing this sub-cohort into four groups (CA 19.9 0.1–2.0, 2.1–9.9, 10.0–19.9, and 20.0–37.0 U/ml), no differences were found among the strata in terms of nodal involvement (SM-Table 3, Supplemental Digital Content 4, http://links.lww.com/JS9/B83).

## Discussion

The present study showed that the standard laboratory cut-off value of CA 19.9 (i.e. 37 U/ml) played a role in the prediction of nodal involvement. Elevations of CA 19.9 levels above the standard laboratory cut-off are significantly associated with a high rate of nodal involvement (>80%).

The investigation of the standard laboratory cut-off of CA 19.9 represents one of the main strengths and innovative aspects of the study, consenting to adopt a user-friendly and cheap tool during the patient staging workup. Other strengths of the study were the large sample size of the investigated cohort, the established pancreatic surgery experience of all the involved centres, and the reliable statistical method.

At multivariable analysis, CA 19.9 was confirmed to be an independent risk factor for nodal involvement. Interestingly, radiological morphology aspects failed to be independent predictors of nodal involvement, confirming the relevance of biological aspects in the discrimination of patients at high risk for worrisome features. As previously reported, pathological tumour information was not included in the construction of the model, with the intent to avoid the risk of immortal biases. Our intent was, in fact, to construct a model based only on preoperative features able to investigate the real value of CA 19.9 in the setting of the therapeutic decision-making process.

Every logarithmic increase of CA 19.9 resulted in doubling the odds of nodal positivity. Furthermore, in cT3 patients, the odds increased up to 6 times, allowing us to consider this subclass of patients as the one who could more significantly benefit from the ability of CA 19.9 to predict lymph node involvement.

Another result observed in the present study was that different CA 19.9 values showed similar abilities to predict lymph node positivity in the different cT and pT sub-classes of patients. This result suggests that the reliable clinical role of the T staging should be further improved in correctly predicting lymph node staging with the integration of CA 19.9.

The negative role of lymph node involvement is well-known in upfront resected R-PDAC^17^. The Heidelberg group demonstrated that a 5-year overall survival (OS) was related to the presence of positive lymph nodes and that CA 19.9 levels impacted OS only in early follow-up. Moreover, the authors reported that CA 19.9 less than 37 U/mL and the number of positive lymph nodes were the only predictors of 5-year disease-free survival^7^.

In line with this evidence, the IAP consensus meeting stated that patients with anatomically R-PDAC with CA 19.9 levels higher than 500 U/ml, or regional lymph node metastases diagnosed by biopsy or suspicious imaging, should be staged as BR-PDAC beyond the classical anatomical criteria^4^.

Similarly, the National Comprehensive Cancer Network (NCCN) guidelines list CA 19.9 levels, large primary tumours, large regional lymph nodes, excessive weight loss, and extreme pain as high-risk features. R-PDAC with these features should be staged as biological BR-PDAC^6^. Notably, no unique cut-off value of CA 19.9 was mentioned, and only radiological dimensional criteria were considered suspicious of lymph node involvement.

The results of the present study confirm these aspects, showing how preoperative CA 19.9 levels should be increasingly central in lymph node status evaluation and, therefore, in R-PDAC management using the standard laboratory cut-off.

Preoperative diagnosis of regional lymph node metastases is essential for optimal patient management, as the staging will shift from upfront resectable to BR-PDAC. Despite recent technological advancements, pancreatic cancer preoperative radiological nodal staging still lacks sensitivity^18^.

Although the impact of the lymph node stage and CA 19.9 levels on survival are well-reported in literature, results on the ability of CA 19.9 to predict lymph node involvement are inconsistent.

In a series published by Kim *et al*.^19^, CA 19.9 could not predict nodal involvement in R-PDAC at cut-offs of 93, 500, and 1000 U/ml.

Bergquist *et al*.^20^ analysed a large series of patients from the National Cancer Data Base (NCDB) and demonstrated that CA 19.9 greater than 37 U/ml harmed OS of early-stage PDAC and that the CA 19.9 elevation was related to significantly higher rates of positive nodes.

These results are in line with the findings of the present study. However, some differences should be noted. First, the main aim of the NCDB study was the OS. Secondly, the study included PDAC regardless of pancreatic tumour location. Thirdly, Bergquist et al. did not perform a matched analysis between CA 19.9 levels and clinical T stages. Lastly, due to the NCDB format, all values of CA 19.9 greater than 98 U/ml were considered together and did not allow further analysis with different CA 19.9 levels^20^. Regarding this last aspect, the present study showed that the percentages of lymph node involvement remain similar when using different CA 19.9 cut-off levels higher than 37 U/ml. This result demonstrated that CA 19.9 greater than 37 U/ml is enough to have a very high risk, around 80%, of lymph node involvement also in radiologically R-PDAC.

In light of our findings, we can postulate that the presence of R-PDAC with CA 19.9 greater than 37 U/ml preoperative staging may suggest a shift from a “radiologically-related” upfront resectable status to a “biologically-related” borderline resectable status. This shift is not without clinical implications, considering the potential clinical value of neo-adjuvant therapies in this setting. In R-PDAC patients, it has been proven that the purpose of neo-adjuvant therapies is to treat nodal and distant micro-metastatic disease and to assess the biological chemosensitivity of the tumours^21^.

The Dutch Pancreatic Cancer Group demonstrated in the PREOPANC Trial that neo-adjuvant therapies could also affect the number of positive lymph nodes as a secondary endpoint^22^.

The potential role of neo-adjuvant therapies should be considered mainly in the sub-group of patients with cT3 status and CA 19.9 greater than 37 U/ml, corresponding to the non-negligible percentage of 7.4% in the present cohort. Obviously, prospectively designed trials are required for confirming the relevance of this approach in this sub-class of patients.

Interestingly, the present study also reported a non-negligible rate of patients with normal CA 19.9 (i.e. CA 19.9 non-secretors) and lymph node involvement. When we investigated the CA 19.9 non-secretors, we were not able to define a specific cut-off of low risk discriminating patients with laboratory normal CA 19.9 values. This group of patients deserves further investigation with the intent to explore other biological markers able to discriminate among non-secretive tumours.

The study presents some limitations. First, the study is retrospective in nature. Second, the re-staging was performed on medical records, and not by a centralised direct imaging review. The 10% of the population that did not express CA 19.9 could play a role in influencing the results. Furthermore, methods used in the preoperative, surgical, and pathology workup could have changed over the study period, especially regarding the margin status analysis.

A relevant aspect to consider is that CA 19.9 can be influenced by other conditions like nutritional status and cholestasis^11,20,23^. As for the albumin levels, an independent predictive ability of both albumin and CA 19.9 was observed in the multivariable model, excluding a potential collinearity phenomenon and, therefore, excluding a specific correlation between these two variables. As for the bilirubin levels, several studies observed that cholestasis may influence CA 19.9 levels^20,23^. However, Anger et al.^23^, logarithmised CA 19.9 and bilirubin to create a model for the calculation of an individually corrected CA 19.9 level in jaundice patients without changing the CA 19.9 staging ability.

In the present study, we observed that both CA 19.9 and bilirubin were independent predictors for N+ status, excluding potential collinearity phenomena. Moreover, a sub-analysis was performed in different classes of bilirubin, showing no impact of the different cholestasis severity strata in terms of lymph node positivity discrimination.

Another limit was the inevitable differences in surgical techniques among the different participating centres. However, we noted that the median number of harvested lymph nodes was similar in the different centres, indicating a similar quality among the different centres involved (i.e. adequate lymph node sampling).

Lastly, the findings of this study are applied to R-PDAC located in the head of the pancreas and cannot be extended to other patient categories.

## Conclusions

The standard laboratory normal cut-off value for CA 19.9 can predict lymph node metastases in radiological R-PDAC. This result can support surgeons and oncologists in the decision-making process in identifying patients with biologically advanced pancreatic cancer that should move from an upfront resectable stage to a borderline resectable stage.

## Ethical approval

The study protocol was approved by the Ethical Committee of Fondazione Policlinico Universitario Campus Bio-Medico, PAR 119/21 (OSS), and the Local Ethics Boards of the involved centres.

## Consent

Due to the retrospective nature of the study, no informed consent was obtained. The Ethics Committee approved the study despite the impossibility of obtaining consent.

A waiver of consent has been granted for access to retrospective data, insofar as outlined within the approved protocol, provided the following requirements are met: (i) The research involves no more than minimal risk to the participants; (ii) The waiver will not adversely affect the rights and welfare of the participant(s); (iii) The research could not practicably be carried out without the waiver or alteration; and (iv) Whenever appropriate, the participants will be provided with additional pertinent information after participation. All medical records or other health-related information must be anonymised (not merely deidentified) at the point of data collection—that is no identifying information such as patient file numbers, names or contact details will be recorded. The data will be aggregated and anonymised in the final reporting. Please refer to UCBM Data Protection Officer (DPO) for authorization to access confidential information for the purposes of research.

## Sources of funding

None.

## Author contribution

Conceptualization: A.C.; Q.L.; T.F.; V.L.V.; R.C. Data curation: A.C.; Q.L.; T.F.; R.C. Formal analysis: A.C.; Q.L. Methodology: A.C.; Q.L.; V.L.V. Project administration: A.C.; Q.L. Supervision: A.C.; Q.L.; H.J.A., U.B., K.C.; B.E.; C.F.; E.J.; N.K.; E.M.P.; S.S.; E.S.; J.S.; A.Z.; N.T.; Q.L.; T.A.; M.B.; R.C.; Y.D.; T.G.; P.G.; M.G.; D.H.; E.F.K.; D.K.; N.L.; R.M.G.; N.N.; G.N.; M.N.; S.R.; M.A.S.;, T.Y.; R.C.; D.C. Validation: A.C.; Q.L.; H.J.A.; U.B.; K.C.; B.E.; C.F.; E.J.; N.K.; E.M.P.; S.S.; E.S.; J.S.; A.Z.; N.T.; Q.L.; T.A.; M.B.; R.C.; Y.D.; T.G.; P.G.; M.G.; D.H.; E.F.K.; D.K.; N.L.; R.M.G.; N.N.; G.N.; M.N.; S.R.; M.A.S.; T.Y.; R.C.; D.C. Writing—original draft: A.C.; Q.L.; T.F.; V.L.V.; R.C. Writing—review and editing: H.J.A.; U.B.; K.C.; B.E.; C.F.; E.J.; N.K.; E.M.P.; S.S.; E.S.; J.S.; A.Z.; N.T.; Q.L.; T.A.; M.B.; R.C.; Y.D.; T.G.; P.G.; M.G.; D.H.; E.F.K.; D.K.; N.L.; R.M.G.; N.N.; G.N.; M.N.; S.R.; M.A.S.; T.Y.; R.C.; D.C.

## Conflicts of interest disclosure

There are not conflicts of interest.

## Research registration unique identifying number (UIN)

We have performed the registration in Clinicaltrials.gov and we are waiting for the UIN. According to the Editors we are going to send you the UIN as soon as possible. The Research Registration Number is: NCT05959213.

## Guarantor

Alessandro Coppola.

## Provenance and peer review

This paper was invited by Professor Lau, Professors Conlon and Professor Coppola for the special series on “Updates on Pancreatic Cancer Treatment”.

## Data statement

Data are available upon reasonable request made to the corresponding author.

## Acknowledgements

The authors acknowledge Benjamin MacCurtin and Laoise Coady for their work, as they pulled the records and constructed the database for inclusion in the study for the Department of HPB Surgery, St. Vincent’s University Hospital, Dublin, Ireland.

## Supplementary Material

SUPPLEMENTARY MATERIAL
