# Supplementary material for: Preoperative carbohydrate antigen 19.9 level predicts lymph node metastasis in resectable adenocarcinoma of the head of the pancreas: a further plea for biological resectability criteria
Source: Int J Surg. 2023 Sep 22;110(10):6092–9. doi: 10.1097/JS9.0000000000000773 (PMC11486984; doi:10.1097/JS9.0000000000000773)
Supplement: SUPPLEMENTARY MATERIAL [file js9-110-6092-s001.pdf]

The STROCSS Guideline		
**Item no.**	**Item description**	**Page No.**
**TITLE**		
1	Title:<br>- The word cohort or cross-sectional or case-controlled is included<br>- The area of focus is described (e.g. disease, exposure/intervention, outcome)<br>- Key elements of study design are stated (e.g. retrospective or prospective)	1
**ABSTRACT**		
2a	Introduction: the following points are briefly described<br>- Background<br>- Scientific Rationale for this study	1
2b	Methods: the following areas are briefly described<br>- Study design (cohort, retro-/prospective, single/multi-centred)<br>- Patient populations and/or groups, including control group, if applicable<br>- Interventions (type, operators, recipients, timeframes)<br>- Outcome measures	1
2c	Results: the following areas are briefly described<br>- Summary data (with statistical relevance) with qualitative descriptions, where appropriate	1
2d	Conclusion: the following areas are briefly described<br>- Key conclusions<br>- Implications to practice<br>- Direction of and need for future research	1
**INTRODUCTION**		
3	Introduction: the following areas are described in full<br>- Relevant background and scientific rationale<br>- Aims and objectives<br>- Research question and hypotheses, where appropriate	2
**METHODS**		
4a	Registration and ethics<br>- Research Registry number is stated, in accordance with the declaration of Helsinki*<br>- All studies (including retrospective) should be registered before submission<br><br>*"*Every research study involving human subjects must be registered in a publicly accessible database before recruitment of the first subject*" (this can be obtained from; ResearchRegistry.com or ClinicalTrials.gov or ISRCTN)	3
4b	Ethical Approval: the following areas are described in full<br>- Necessity for ethical approval<br>- Ethical approval, with relevant judgement reference from ethics committees<br>- Where ethics was unnecessary, reasons are provided	3
4c	Protocol: the following areas are described comprehensively<br>- Protocol (*a priori* or otherwise) details, with access directions<br>- If published, journal mentioned with the reference provided	3

4d	Patient involvement in Research<br>- Describe how, if at all, patients were involved in study design e.g. were they involved on the study steering committee, did they provide input on outcome selection, etc.	3,4
5a	Study Design: the following areas are described comprehensively<br>- 'Cohort' study is mentioned<br>- Design (e.g. retro-/prospective, single/multi-centred)	3,4
5b	Setting: the following areas are described comprehensively<br>- Geographical location<br>- Nature of institution (e.g. academic/community, public/private)<br>- Dates (recruitment, exposure, follow-up, data collection)	N.A.
5c	Cohort Groups: the following areas are described in full<br>- Number of groups<br>- Division of intervention between groups	N.A.
5d	Subgroup Analysis: the following areas are described comprehensively<br>- Planned subgroup analyses<br>- Methods used to examine subgroups and their interactions	N.A.
6a	Participants: the following areas are described comprehensively<br>- Eligibility criteria<br>- Recruitment sources<br>- Length and methods of follow-up	3,4
6b	Recruitment: the following areas are described comprehensively<br>- Methods of recruitment to each patient group<br>- Period of recruitment	3,4
6c	Sample Size: the following areas are described comprehensively<br>- Margin of error calculation<br>- Analysis to determine study population<br>- Power calculations, where appropriate	4,5
**Intervention and Considerations**		
7a	Pre-intervention Considerations: the following areas are described comprehensively<br>- Patient optimisation (pre-surgical measures)<br>- Pre-intervention treatment (hypothermia/-volaemia/-tension; ICU care; bleeding problems; medications)	N.A.
7b	Intervention: the following areas are described comprehensively<br>- Type of intervention and reasoning (e.g. pharmacological, surgical, physiotherapy, psychological)<br>- Aim of intervention (preventative/therapeutic)<br>- Concurrent treatments (antibiotics, analgaesia, anti-emetics, NBM, VTE prophylaxis)<br>- Manufacturer and model details where applicable	N.A.
7c	Intra-Intervention Considerations: the following areas are described comprehensively<br>- Administration of intervention (location, surgical details, anaesthetic, positioning, equipment needed, preparation, devices, sutures, operative time)<br>- Pharmacological therapies include formulation, dosages, routes and durations<br>- Figures other media are used to illustrate	N.A.

7d	Operator Details: the following areas are described comprehensively<br>- Training needed<br>- Learning curve for technique<br>- Specialisation and relevant training	N.A.
7e	Quality Control: the following areas are described comprehensively<br>- Measures taken to reduce variation<br>- Measures taken to ensure quality and consistency in intervention delivery	N.A.
7f	Post-Intervention Considerations: the following areas are described comprehensively<br>- Post-operative instructions and care<br>- Follow-up measures<br>- Future surveillance requirements (e.g. imaging, blood tests)	N.A.
8	Outcomes: the following areas are described comprehensively<br>- Primary outcomes, including validation, where applicable<br>- Definitions of outcomes<br>- Secondary outcomes, where appropriate<br>- Follow-up period for outcome assessment, divided by group	N.A.
9	Statistics: the following areas are described comprehensively<br>- Statistical tests, packages/software used, and interpretation of significance<br>- Confounders and their control, if known<br>- Analysis approach (e.g. intention to treat/per protocol)<br>- Sub-group analysis, if any	4,5
**RESULTS**		
10a	Participants: the following areas are described comprehensively<br>- Flow of participants (recruitment, non-participation, cross-over and withdrawal, with reasons)<br>- Population demographics (prognostic features, relevant socioeconomic features, and significant numerical differences)	5,6,7,8
10b	Participant Comparison: the following areas are described comprehensively<br>- Table comparing demographic included<br>- Differences, with statistical relevance<br>- Any group matching, with methods	5,6,7,8
10c	Intervention: the following areas are described comprehensively<br>- Changes to interventions, with rationale and diagram, if appropriate<br>- Learning required for interventions<br>- Degree of novelty for intervention	NA
11a	Outcomes: the following areas are described comprehensively<br>- Clinician-assessed and patient-reported outcomes for each group<br>- Relevant photographs and imaging are desirable<br>- Confounders to outcomes and which are adjusted	N.A.
11b	Tolerance: the following areas are described comprehensively<br>- Assessment of tolerance<br>- Loss to follow up, with reasons (percentage and fraction)<br>- Cross-over with explanation	N.A.
11c	Complications: the following areas are described comprehensively<br>- Adverse events described<br>- Classified according to Clavien-Dindo classification*	N.A.

	- Mitigation for adverse events (blood loss, wound care, revision surgery should be specified)<br><br>*Dindo D, Demartines N, Clavien P-A. Classification of Surgical Complications. A New Proposal with Evaluation in a Cohort of 6336 Patients and Results of a Survey. Ann Surg. 2004; 240(2): 205-213	
12	Key Results: the following areas are described comprehensively<br>- Key results, including relevant raw data<br>- Statistical analyses with significance	7
**DISCUSSION**		
13	Discussion: the following areas are described comprehensively<br>- Conclusions and rationale<br>- Reference to relevant literature<br>- Implications to clinical practice<br>- Comparison to current gold standard of care<br>- Relevant hypothesis generation	8,9,10,11
14	Strengths and Limitations: the following areas are described comprehensively<br>- Strengths of the study<br>- Limitations and potential impact on results<br>- Assessment of bias and management	11,12
15	Implications and Relevance: the following areas are described comprehensively<br>- Relevance of findings and potential implications to clinical practice are detailed<br>- Future research that is needed is described, with study designs detailed	10,11
**CONCLUSION**		
16	Conclusions:<br>- Key conclusions are summarised<br>- Key directions for future research are summarised	12
**DECLARATIONS**		
17a	Conflicts of interest<br>- Conflicts of interest, if any, are described	Title page
17b	Funding<br>- Sources of funding (e.g. grant details), if any, are clearly stated	Title page
