# Supplementary material for: Preoperative carbohydrate antigen 19.9 level predicts lymph node metastasis in resectable adenocarcinoma of the head of the pancreas: a further plea for biological resectability criteria
Source: Int J Surg. 2023 Sep 22;110(10):6092–9. doi: 10.1097/JS9.0000000000000773 (PMC11486984; doi:10.1097/JS9.0000000000000773)
Supplement: SUPPLEMENTARY MATERIAL [file js9-110-6092-s002.docx]

**SM-Table 1.** Missing data in the entire population (N=2,034).

| **Variables** | **Missing data (n)** | **(%)** |
| --- | --- | --- |
| **Age** | 0 | 0.0 |
| **Sex** | 0 | 0.0 |
| **BMI≥30** | 0 | 0.0 |
| **T2DM** | 0 | 0.0 |
| **ASA Score 3-4** | 0 | 0.0 |
| **Preoperative CA 19.9** | 0 | 0.0 |
| **Preoperative albumin** | 40 | 2.0 |
| **Preoperative total bilirubin** | 52 | 2.6 |
| **Tumor size at imaging** | 0 | 0.0 |
| **Tumor size at pathology** | 0 | 0.0 |
| **Vascular resection** | 0 | 0.0 |
| **Vascular resection type** | 0 | 0.0 |
| **R1-R2** | 0 | 0.0 |
| **Tumor grading** | 0 | 0.0 |
| **Harvested nodes** | 0 | 0.0 |
| **Positive nodes** | 0 | 0.0 |
| **Abbreviations:** BMI, body mass index; T2DM, type-2 diabetes mellitus; ASA, American Society of Anesthesiologists. | | |
