# Supplementary material for: Preoperative carbohydrate antigen 19.9 level predicts lymph node metastasis in resectable adenocarcinoma of the head of the pancreas: a further plea for biological resectability criteria
Source: Int J Surg. 2023 Sep 22;110(10):6092–9. doi: 10.1097/JS9.0000000000000773 (PMC11486984; doi:10.1097/JS9.0000000000000773)
Supplement: SUPPLEMENTARY MATERIAL [file js9-110-6092-s003.docx]

**SM-Table 2.** Pathologic lymph nodes positivity results according to clustered preoperative CA 19.9 levels in the subclasses of patients according to the total bilirubin value at the same time of CA 19.9 measurement.

| **CA 19.9 U/mL** | **n** | **N+** | **N2** |
| --- | --- | --- | --- |
| **Total bilirubin 0.10-1.50 mg/dL** | | | |
| ≤37 | 378 | 243 (64.3) | 109 (28.8) |
| 38-249 | 376 | 295 (78.5) | 134 (35.6) |
| 250-499 | 106 | 87 (82.1) | 45 (42.5) |
| 500-999 | 87 | 70 (80.5) | 42 (48.3) |
| ≥1,000 | 116 | 94 (81.0) | 57 (49.1) |
| p-value |  | <0.001 | <0.001 |
| **Total bilirubin 1.51-3.00 mg/dL** | | | |
| ≤37 | 79 | 58 (73.4) | 36 (45.6) |
| 38-249 | 128 | 105 (82.0) | 51 (39.8) |
| 250-499 | 28 | 25 (89.3) | 12 (42.9) |
| 500-999 | 33 | 29 (87.9) | 16 (48.5) |
| ≥1,000 | 57 | 49 (86.0) | 27 (47.4) |
| p-value | - | 0.18 | 0.82 |
| **Total bilirubin > 3.00 mg/dL** | | | |
| ≤37 | 139 | 102 (73.4) | 49 (35.3) |
| 38-249 | 205 | 168 (82.0) | 88 (42.9) |
| 250-499 | 86 | 69 (80.2) | 35 (40.7) |
| 500-999 | 73 | 62 (84.9) | 37 (50.7) |
| ≥1,000 | 143 | 120 (83.9) | 83 (58.0) |
| p-value | - | 0.15 | 0.002 |
| **Abbreviations:** n, number; N+, nodal positivity at pathology; N2, nodal 2 stage. | | | |
