# Supplementary material for: Preoperative carbohydrate antigen 19.9 level predicts lymph node metastasis in resectable adenocarcinoma of the head of the pancreas: a further plea for biological resectability criteria
Source: Int J Surg. 2023 Sep 22;110(10):6092–9. doi: 10.1097/JS9.0000000000000773 (PMC11486984; doi:10.1097/JS9.0000000000000773)
Supplement: SUPPLEMENTARY MATERIAL [file js9-110-6092-s004.docx]

**SM-Table 3.** Pathologic lymph nodes positivity results according to clustered preoperative CA 19.9 levels: only patients with CA19.9 ≤37 U/mL.

| **CA 19.9 U/mL** | **n** | **N+** | **N2** |
| --- | --- | --- | --- |
| 0.1-2.0 | 113 | 76 (67.3) | 37 (32.7) |
| 2.1-9.9 | 131 | 88 (67.2) | 31 (31.3) |
| 10.0-19.9 | 155 | 97 (62.6) | 55 (35.5) |
| 20.0-37.0 | 197 | 142 (72.1) | 61 (31.0) |
| p-value | - | 0.31 | 0.82 |
| **Abbreviations:** n, number; N+, nodal positivity at pathology; N2, nodal 2 stage. | | | |
